# Supplementary material for: Light-driven nucleation, growth, and patterning of biorelevant crystals using resonant near-infrared laser heating
Source: Nat Commun. 2023 Oct 10;14:6350. doi: 10.1038/s41467-023-42126-4 (PMC10564937; doi:10.1038/s41467-023-42126-4)
Supplement: Supplementary file 1 — Supplementary Information [file 41467_2023_42126_MOESM1_ESM.pdf]

# Supplementary Information

**Light-driven nucleation, growth, and patterning of biorelevant crystals using resonant near-infrared laser heating**

*Marloes H. Bistervels[1], Balázs Antalicz[1], Marko Kamp[1], Hinco Schoenmaker[1], Willem L. Noorduin[1,2]*

[1] Science Park 104, 1098 XG Amsterdam, The Netherlands
[2] Van't Hoff Institute for Molecular Sciences, University of Amsterdam, Amsterdam 1090 GD, The Netherlands
E-mail: noorduin@amolf.nl

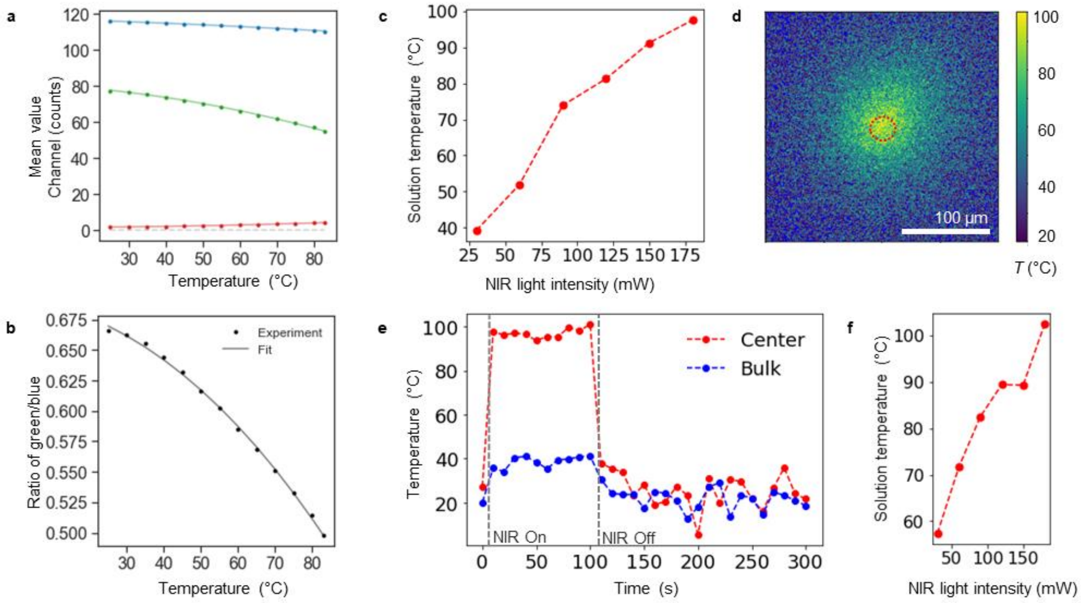


**Supplementary Figure 1. Interfacial temperature measurements at the center of the heating NIR beam.** To monitor spatially resolved interfacial temperature changes upon NIR laser irradiation, we developed a fluorescence-based experimental method that utilizes the photoacid 8-hydroxypyrene-1,3,6-trisulfonic trisodium salt (HPTS). HPTS has two emission bands, centered at 440 and 510 nm. These bands almost perfectly overlap with the green (G) and blue (B) channels of the CMOS sensor in our transmission microscope, which allows monitoring their ratios without a spectrometer. To excite HPTS, we illuminate the sample cell from the same side as the NIR laser diode, using a commercial, low-intensity 400 nm UV LED. To evaluate local temperature changes induced by the NIR laser heating, we construct a calibration curve based on G:B ratios. To this aim, we heat the HPTS solution in the reaction cell with a bath and circulation thermostat (Huber CC-K6) to 83 °C under illumination of a 400 nm LED and record the RGB channels. **(a)** Mean values of blue, green, and red channel of the color CMOS camera upon externally controlled heating. Blue, green, and red dots and lines represent experimental and fitted data respectively of the different blue, green, and red channels. **(b)** Calibration curve of the RGB ratio values with the absolute temperature. Data points are fitted with a second order polynomial. **(c)** Solution temperature upon NIR light intensity in the beam area. **(d)** Spatial temperature distribution upon 180 mW NIR irradiation. The dashed red circle represents the NIR focal spot. **(e)** Time-dependent solution temperature upon with 180 mW NIR irradiation, at the beam center (red dots) and in the bulk of the solution, ca. 150 µm away from the NIR focal point (blue dots). The NIR was manually turned on and off after 10 and 110 seconds, respectively, indicated by the grey dashed lines. **(f)** Solution temperature upon NIR irradiation on an ITO coated substrate.

**Supplementary note 1. Interfacial temperature measurements at the center of the heating NIR beam.**

The choice of wavelength (400 nm) allows us to excite HPTS at its absorption maximum, ($\epsilon_{\text{att}} \approx 20000 \frac{l}{\text{mol cm}^{-1}}$) (1). For the UV light, this yields a penetration depth (1/e transmission, ca. 37%) of $d^{\text{UV}} = \frac{\log e}{\epsilon_{\text{att}}\, c} \approx 43\ \mu m$ (2); which is much shorter compared to the ≈370 um penetration depth of the NIR beam (1,2). Because $d^{\text{UV}} \ll d^{\text{NIR}}$, we essentially sample the interfacial temperature of the heated solution. We find that the temperature a) changes within seconds, and b) increases approximately linearly with NIR intensity, and c) reaches ca. 90 ± 5 °C upon a NIR heating of 180 mW (Supplementary Figure 1c-e). For reaction cells built with ITO coated substrates, we find a higher maximum temperature change upon NIR light intensity, reaching ca. 110 ± 10 °C upon a NIR light intensity of 180 mW (Supplementary Figure 1f). Note that the irradiated part of the ITO coated substrate will be even higher in temperature than the measured solution due to additional heating (Supplementary Figure 3). Using the thermocouple, the bulk solution measured at ca. 1 mm from the heating spot rises only from ca. 26 °C to 30 °C during 17 h of NIR heating.

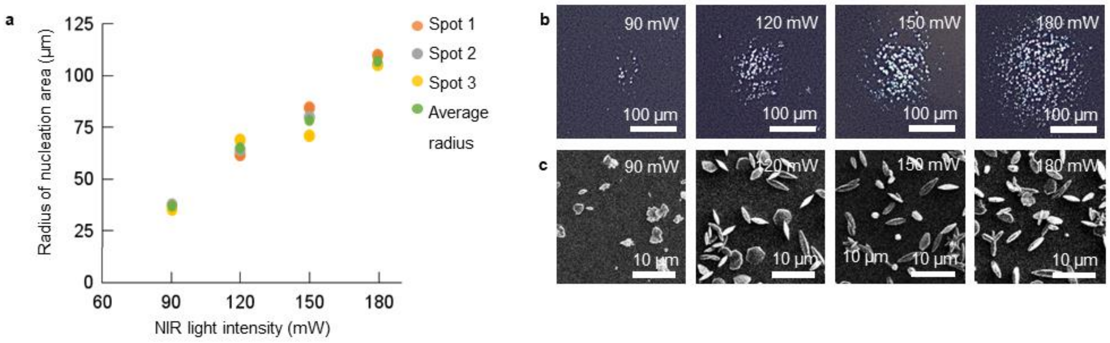


**Supplementary Figure 2. Nucleation of $CaCO_3$ upon 45 seconds of irradiation with different NIR light intensities.** To investigate how the NIR light intensity determines the nucleation rate of CaCO3, four reaction cells with a freshly prepared precursor solution of fixed concentrations (1.5 mM CaCl2, 1 mM Na2CO3, 300 mM NaCl, pH 10.7) are prepared and a NIR light beam is focused with different light intensities on the substrates of the reaction cells. Per reaction cell, one NIR light intensity (90, 120, 150, or 180 mW) with which we subsequentially irradiate three light spots is used, each for 45 s. After the NIR light irradiation, the cell with is directly washed with water and ethanol and let it dry in air. To analyze and compare the induced nucleation upon the irradiation with different light intensity, we use in-situ monitoring, light microscope imaging, and SEM imaging. For the freshly prepared solutions, we find the nucleation of mainly vaterite and aragonite in a small radius (35-110 µm) of the irradiated area with all light intensities. **(a)** Plot of the area in which nucleation is observed upon NIR light intensity. Within the regime of 90-180 mW, the area where nucleation is observed increases almost linearly for increasing light intensities. **(b)** Light microscopy images of the NIR light induced nucleation. The results indicate a light intensity offset with respect to nucleation, since with 90 mW NIR light intensity almost no nucleation is observed. **(c)** SEM images of NIR light induced nucleation at the center of the NIR irradiated area. For light intensities of 120 mW and higher, the nucleation density at the center of the light spot does not significantly differ within this time span of 45 s.

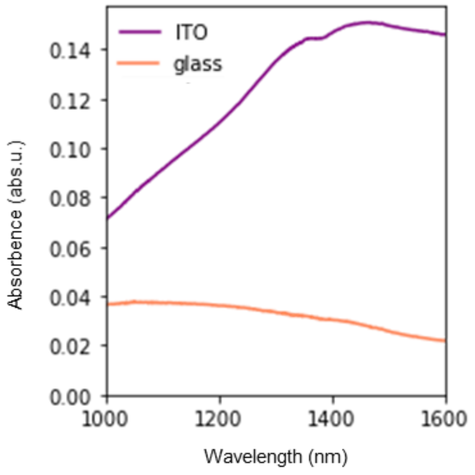


**Supplementary Figure 3. NIR absorption of glass and ITO.** Steady-state external absorbance measurements of glass (orange line) and ITO-coated glass (purple line) in the range of 1000-1600 nm using a LAMBDA 750 UV/vis/NIR spectrophotometer (Perkin Elmer), equipped with a deuterium and tungsten excitation source, an InGaAs detector, and an integrating sphere.

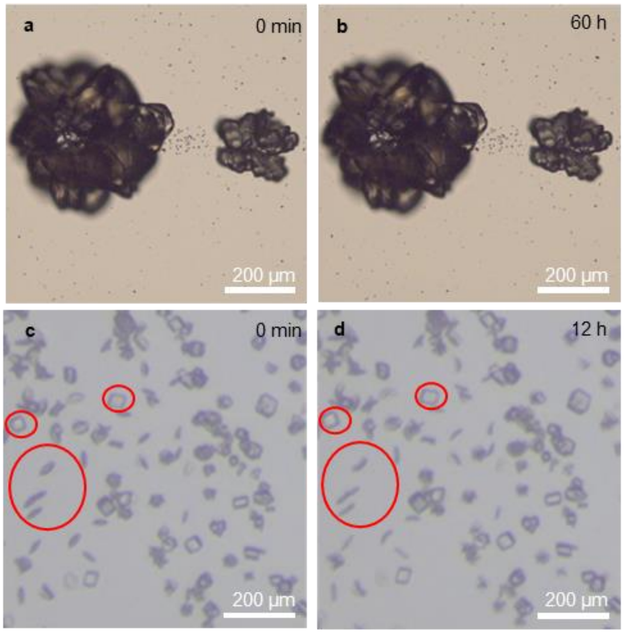


**Supplementary Figure 4. Dissolution test**. Monitor of NIR light induced $CaCO_3$ when placed outside the NIR light spot. To test if dissolution of NIR light induced crystals occurs when placed outside the NIR light spot, we closely monitor a reaction cell filled with a 2 days old $CaCO_3$ precursor solution (0.5 mM $CaCl_2$, 1 mM $Na_2CO_3$, 400 mM NaCl, pH 10.7) with and without NIR irradiation. First, we induce the nucleation and growth of $CaCO_3$ upon high intensity (180 mW). We continue NIR light irradiation until no further growth is observed (48 h). We turn off the NIR laser light, but we continue to monitor the grown crystals (60 h). **(a)** Light microscopy image of NIR light induced $CaCO_3$ crystals at $t$ = 0 min, directly, placed outside the NIR light spot and **(b)** $t$ = +60 h. No dissolution is observed dissolution for neither the large grown crystals nor small nucleated calcite-like crystals, indicating that the NIR light grown calcite crystals remain stable and that the growth solution is only slightly undersaturated at room temperature after the NIR irradiation. To evaluate the thermodynamic stability between polymorphs after irradiation, we induce the nucleation of a mixture of calcite and vaterite with a freshly prepared precursor solution which contains rather high precursor concentrations (5 mM $CaCl_2$, 1 mM $Na_2CO_3$, 100 mM NaCl pH 10.7). We irradiate for several hours with high light intensity to induce nucleation and growth of mixed $CaCO_3$ (180 mW for 3 h). **(c)** Light microscopy image of NIR light induced vaterite and calcite crystals at (i) $t$ = 0 min, directly placed outside the NIR light spot, and **(d)** $t$ = +12 h. Red circles point out the dissolution of vaterite and growth of calcite. We observe slow dissolution of small vaterite crystals and further growth of calcite crystals which indicates that the light induced small vaterite is thermodynamic less stable than calcite.

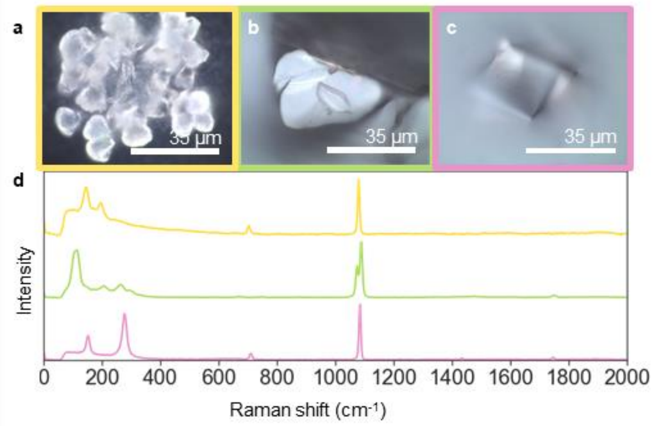


**Supplementary Figure 5. Raman spectra of NIR light induced $CaCO_3$ polymorphs. (a)** Light microscope imaging of the probed area for Raman spectroscopy of $CaCO_3$ grown in presence of $MgCl_2$, **(b)** $CaCO_3$ grown in presence of NaCl without equilibration time, and **(c)** $CaCO_3$ grown in presence of NaCl after 3 h of equilibration time. **(d)** Raman spectra of $CaCO_3$ indicated by colored boxes and indices corresponding with (a-c), top (yellow) aragonite, middle (green) vaterite, and bottom (pink) calcite.

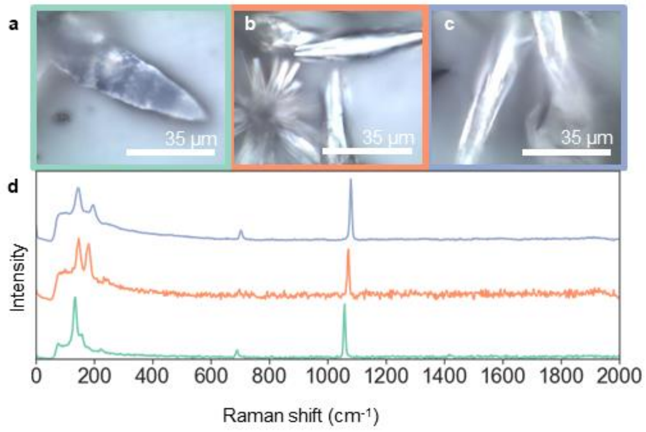


**Supplementary Figure 6. Raman spectra of NIR light induced $MCO_3$ crystals. (a)** Light microscope imaging of the probed area for Raman spectroscopy of $BaCO_3$, **(b)** $SrCO_3$, and **(c)** $CaCO_3$. **(d)** Raman spectra of the $MCO_3$ crystals indicated by colored boxes and indices corresponding with (a-c), top (blue) $CaCO_3$, middle (orange) $SrCO_3$, and bottom (green) $BaCO_3$.

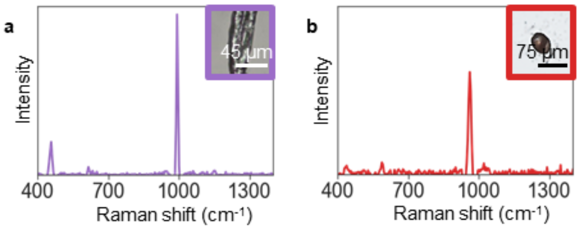


**Supplementary Figure 7. Raman spectra of NIR light induced $SrSO_4$ and calcium phosphate crystals. (a)** $SrSO_4$, and **(b)** calcium phosphate crystals with the light microscope image of the probed area in the inset.

**Supplementary references**


1. G. M. Hale and M. R. Querry, Optical constants of water in the 200-nm to 200-µm wavelength region, Appl. Opt. 12, 555-563 (1973).
2. R. Sabnis, Handbook of Fluorescent Dyes and Probes, Chapter 90 (Wiley, 2015).
