# Supplementary material for: Light-driven nucleation, growth, and patterning of biorelevant crystals using resonant near-infrared laser heating
Source: Nat Commun. 2023 Oct 10;14:6350. doi: 10.1038/s41467-023-42126-4 (PMC10564937; doi:10.1038/s41467-023-42126-4)
Supplement: Supplementary file 3 — Description of Additional Supplementary Files [file 41467_2023_42126_MOESM3_ESM.pdf]

Description of Additional Supplementary Files

File Name: Supplementary Movie 1

Description: **Movie NIR light induced nucleation and growth of $CaCO_3$ crystals**

In this movie, the nucleation and growth of CaCO3 crystals positioned in a line (described in the main text) are presented. The red dashed circle represents the location of NIR light spot. The total movie consists of 453 frames that cover a time span of 61 h real-time growth. Due to the long timespan the video contains some time jumps. The real-time of crystallization per crystal as numbered in the movie is (times of nucleation are an estimation):

1. 16 h (growth 120 mW, nucleation within first minute with 180 mW)
2. 6 h (growth 120 mW, nucleation within first hour as well with 120 mW)
3. 14 h (growth 130 mW, nucleation within first hour with 140 mW)
4. 9 h (130 mW, nucleation within first 2 h as well with 130 mW)
5. 17 h (120 mW, nucleation within first 3 h as well with 120 mW)
